# Time-Optimal Trajectory Planning and Tracking for Autonomous Vehicles

**DOI:** 10.3390/s24113281

**Published:** 2024-05-21

**Authors:** Jun-Ting Li, Chih-Keng Chen, Hongbin Ren

**Affiliations:** 1 Department of Vehicle Engineering, National Taipei University of Technology, Taipei 10604, Taiwan; t112669009@ntut.org.tw; 2 School of Mechanical Engineering, Beijing Institute of Technology, Beijing 100081, China; renhongbin2106@126.com

**Keywords:** autonomous racing, trajectory planning, trajectory tracking, NMPC

## Abstract

This article presents a hierarchical control framework for autonomous vehicle trajectory planning and tracking, addressing the challenge of accurately following high-speed, at-limit maneuvers. The proposed time-optimal trajectory planning and tracking (TOTPT) framework utilizes a hierarchical control structure, with an offline trajectory optimization (TRO) module and an online nonlinear model predictive control (NMPC) module. The TRO layer generates minimum-lap-time trajectories using a direct collocation method, which optimizes the vehicle’s path, velocity, and control inputs to achieve the fastest possible lap time, while respecting the vehicle dynamics and track constraints. The NMPC layer is responsible for precisely tracking the reference trajectories generated by the TRO in real time. The NMPC also incorporates a preview algorithm that utilizes the predicted future travel distance to estimate the optimal reference speed and curvature for the next time step, thereby improving the overall tracking performance. Simulation results on the Catalunya circuit demonstrated the framework’s capability to accurately follow the time-optimal raceline at an average speed of 116 km/h, with a maximum lateral error of 0.32 m. The NMPC module uses an acados solver with a real-time iteration (RTI) scheme, to achieve a millisecond-level computation time, making it possible to implement it in real time in autonomous vehicles.

## 1. Introduction

Recent autonomous driving research has aimed to achieve Level 5 self-driving capabilities [1,2] for production vehicles, which requires path tracking controllers to precisely coordinate the throttle, brakes, and steering to control vehicles along predefined paths and velocity profiles. This task becomes particularly challenging when vehicle dynamics approach handling limits during high-speed maneuvers, such as lane changes or obstacle avoidance [3]. Consequently, these challenges have propelled autonomous racing to the forefront as a popular research topic [4], as it involves controlling high-performance vehicles under highly nonlinear dynamics with short response times. If vehicles can be reliably controlled within or even beyond their handling limits in such extreme racing or drifting scenarios [5], this could enhance their maneuverability when encountering emergency situations on normal roads, thereby improving overall driving safety. The core of autonomous racing lies in trajectory optimization, which involves planning the time-optimal racing line for a given parameterized racetrack. This process typically entails a comprehensive consideration of various vehicle performance factors and optimization objectives, including drivetrain configuration [6], tire–road friction coefficients [7], active rear-axle steering [8], gear ratios, aerodynamics, roll stiffness, suspension characteristics, and other variable parameters [9,10,11,12]. Trajectory optimization problems are typically discretized via the direct collocation method and solved using nonlinear programming (NLP) solvers. The key advantage of this approach is the capability to handle high-order nonlinear dynamic system models and complex constraints, while yielding fully converged optimal solutions.

Hierarchical control architectures have been developed to enable autonomous racing cars to accurately follow time-optimal reference trajectories planned in real time. By separating the control task into high and low levels with different prediction horizons and sampling times, the hierarchical approach increases the controller’s look-ahead capability, while maintaining a computationally manageable size for NLP problems [13]. The hierarchical control scheme in [14] consisted of a long prediction horizon NMPC with a point-mass model and a short horizon NMPC with a high-fidelity vehicle model. This structure allowed for real-time feasibility, without excessively simplifying the vehicle model. Furthermore, Srinivasan et al. [15] implemented a hierarchical control structure in a race car, comprising a 200 Hz low-level controller and a 40 Hz high-level NMPC with an optimized torque vectoring strategy. This strategy minimized the model mismatch between planning and control and verified the ability to outperform professional human drivers in racing scenarios.

This research presents TOTPT, a unified hierarchical optimization framework for controlling autonomous vehicles at their handling limits. It utilizes a TRO layer that plans the time-optimal raceline and velocity offline, and an NMPC layer that tracks the references online. The open-source code for the TOTPT framework is available at the online repository (https://github.com/zlijunting/TOTPT, accessed on 10 May 2024).

This study primarily focused on the development of the following aspects:

- A unified vehicle modeling and control framework: The framework employs a consistent four-wheel double-track vehicle model across both the offline TRO and online NMPC layers. This unified modeling approach ensures coherent predictive behavior and enables efficient integration between the planning and control modules. Furthermore, proper scaling of variables and constraints, combined with well-defined problem formulations, facilitates rapid convergence of the optimization solvers in both layers.
- Tailored tire modeling: Both the TRO and NMPC layers incorporate tire ellipse constraints on all four wheels, to enhance driving stability. The TRO layer utilizes hard tire ellipse constraints to fully utilize tire grip. In contrast, the NMPC layer imposes soft tire ellipse constraints using a pure slip tire model, which is more suitable for the multiple shooting scheme and convergence requirements of NMPC.
- An adaptive reference preview algorithm: The reference trajectory adapts to the vehicle’s speed. Specifically, it utilizes the travel distance and speed predicted from the NMPC to estimate the optimal path station and corresponding reference speed and curvature for the next time step. This approach improves the trajectory tracking performance by fully leveraging the model’s predictive capabilities.

This paper is organized as follows: Section 2 describes the kinematic modeling of the vehicle relative to the reference path and the dynamics modeling for path tracking. Section 3 details the 3-DOF double-track vehicle dynamics model, covering the load transfer, wheel torques, nonlinear tire models, and vehicle constraints. Section 4 provides an overview of the hierarchical control scheme, with the offline TRO module generating optimal trajectories and the online modules handling error evaluation, path preview, and NMPC trajectory tracking. Section 5 formulates the TRO problem for offline trajectory planning, using the direct collocation method to discretize the problem into an NLP. Section 6 presents the formulation of the NMPC problem for online trajectory tracking, including the prediction model, discretization method, cost function, and constraints. Additionally, a reference preview algorithm is proposed. Section 7 presents the time-optimal trajectory planned using TRO, along with the trajectory tracking results obtained using NMPC. The path tracking performance, tire workloads, and execution efficiency are also analyzed.

## 2. Track Modeling

**Figure 1:**
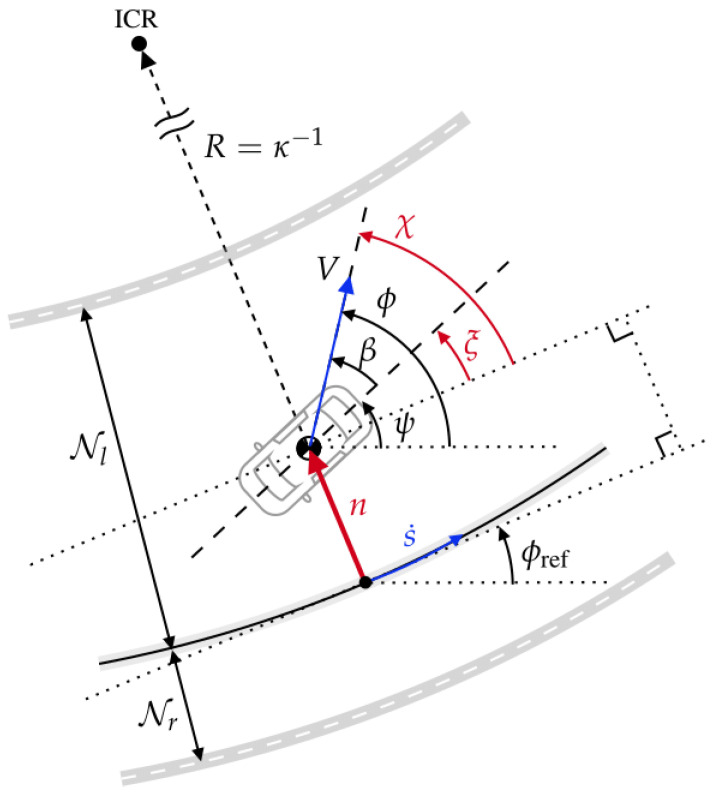
Kinematic relationship between the vehicle and the reference path.

A scheme diagram of the path tracking model is illustrated in Figure 1, where *R* is the distance from the vehicle CG to the instantaneous center of rotation (ICR), $\kappa =d\phi_{\mathrm{ref}}{\left(ds\right)}^{-1}$ is the path curvature at a given path station *s*, $\phi_{\mathrm{ref}}$ is the reference course angle, and $N_{l}\in R^{+}$ and $N_{r}\in R^{+}$ are the normal distances from the path to the left and right track boundaries. The vehicle moves in the direction defined by the vehicle’s course angle ϕ, with a velocity of *V*. The relative states between the vehicle and the path include the normal distance from the vehicle’s CG to the nearest point on the path *n*, the relative heading $\xi =\psi -\phi_{\mathrm{ref}}$, and relative course $\chi =\phi -\phi_{\mathrm{ref}}=\xi +\beta$.

The lateral deviation and relative heading are selected as the primary path tracking states, then the path tracking dynamics are described by the following equations:

(1) $$\begin{array}{cc} \dot{n} & =V\sin(\chi ), \end{array}$$

(2) $$\begin{array}{cc} \dot{\xi } & =\gamma -\frac{d\phi_{\mathrm{ref}}}{ds}\frac{ds}{dt} \end{array}$$

(3) $$\begin{array}{cc} \; & =\gamma -\kappa \dot{s}, \end{array}$$

where $\dot{s}$ is the vehicle velocity projected onto the direction of the reference course at path

(4) $$\begin{array}{c} \dot{s}=\frac{V\cos(\chi )}{1-\kappa n}. \end{array}$$

The ‘distance traveled’ is used as the independent variable for the TRO [9]. Subsequently, the dependent variable, i.e., time traveled for a given path grid, can be obtained using the factor $S_{f}$, as defined by

(5) $$\begin{array}{c} \frac{dt}{ds}=\frac{1-\kappa n}{V\cos(\chi )}≕S_{f}. \end{array}$$

### Track Constraint

The lateral deviation of the vehicle is limited, to ensure that it remains within the track boundaries,

(6) $$\begin{array}{c} -N_{r}+\left(\frac{w_{t}}{2}+w_{s}\right)\leq n\leq N_{l}-\left(\frac{w_{t}}{2}+w_{s}\right), \end{array}$$

where $w_{t}$ is the vehicle track width, and $w_{s}=0.2\;m$ is the preserved safety distance.

## 3. Vehicle Modeling

The choice of vehicle models for the path-following problem is diverse [16]. Considering vehicle control near handling limits during racing maneuvers, the selected model must capture the coupling of longitudinal and lateral motions, as well as the nonlinear behavior of tire forces. In this research, a 3-degree-of-freedom (DOF) double-track vehicle model is employed to address both the TRO and NMPC problems. Aerodynamic drag and lift forces are included, while suspension dynamics are ignored. A rear-wheel drive (RWD) sports car with dual-motor is utilized as the test vehicle in this study, and the vehicle parameters are specified in Table A1.

### 3.1. Double-Track Vehicle Model

**Figure 2:**
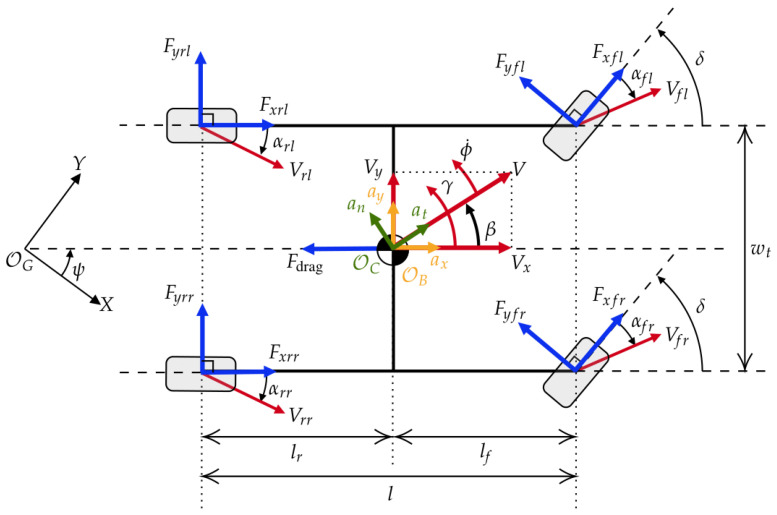
Diagram of the double-track vehicle model.

Figure 2 shows the layout of the double-track vehicle model, with the vehicle heading at yaw angle ψ from the *x*-axis of the predefined global coordinates $O_{G}$. The accelerations $a_{x}$ and $a_{y}$ are located at the vehicle body coordinate $O_{B}$, and $a_{t}$ and $a_{n}$ are located at the vehicle course coordinate $O_{C}$. These accelerations are expressed by the following equations:

(7) $$\begin{array}{ccc} a_{x}= & \frac{1}{m_{v}}\left[F_{xf}\cos\left(\delta \right)-F_{yf}\sin\left(\delta \right)+F_{xr}-F_{\mathrm{drag}}\right], & \; \end{array}$$

(8) $$\begin{array}{ccc} a_{y}= & \frac{1}{m_{v}}\left[F_{yf}\cos\left(\delta \right)+F_{xf}\sin\left(\delta \right)+F_{yr}\right], & \; \end{array}$$

(9) $$\begin{array}{cc} a_{t}= & \frac{1}{m_{v}}\left[F_{xf}\cos\left(\delta -\beta \right)-F_{yf}\sin\left(\delta -\beta \right)+F_{xr}\cos\left(\beta \right)+F_{yr}\sin\left(\beta \right)-F_{\mathrm{drag}}\cos\left(\beta \right)\right], \end{array}$$

(10) $$\begin{array}{cc} a_{n}= & \frac{1}{m_{v}}\left[F_{xf}\sin\left(\delta -\beta \right)+F_{yf}\cos\left(\delta -\beta \right)-F_{xr}\sin\left(\beta \right)+F_{yr}\cos\left(\beta \right)+F_{\mathrm{drag}}\sin\left(\beta \right)\right], \end{array}$$

where $m_{v}$ is the vehicle mass, δ is the front wheel steering angle, $F_{xf/l}$ is the total longitudinal tire force at the front/rear axle, $F_{yf/l}$ is the total lateral tire force at the front/rear axle, and $F_{\mathrm{drag}}=\frac{1}{2}\rho C_{d}AV_{x}^{2}$ is the aerodynamic drag force, where ρ is the air density, $C_{d}$ is the drag coefficient, *A* is the vehicle frontal area, and $V_{x}$ is the longitudinal vehicle speed.

The yaw moment about the center of gravity (CG) of the vehicle is

(11) $$\begin{array}{cc} M_{z} & =l_{f}F_{yf}\cos\left(\delta \right)+l_{f}F_{xf}\sin\left(\delta \right)-l_{r}F_{yr}\\ \; & +\frac{w_{t}}{2}\left[\left(F_{yfl}-F_{yfr}\right)\sin\left(\delta \right)+\left(F_{xfr}-F_{xfl}\right)\cos\left(\delta \right)+\left(F_{xrr}-F_{xrl}\right)\right], \end{array}$$

where $l_{f}$ and $l_{r}$ are the distances from CG to the front and rear axles.

Similarly to the vehicle models in [6,7,17,18], the total velocity *V*, sideslip angle β, and yaw rate γ are selected as the state variables; then, the following equations are used to describe the planar dynamics of the vehicle:

(12) $$\begin{array}{ccc} \dot{V} & =a_{t}, & \; \end{array}$$

(13) $$\begin{array}{cc} \dot{\beta } & =\dot{\phi }-\dot{\psi }=\frac{a_{n}}{V}-\gamma , \end{array}$$

(14) $$\begin{array}{ccc} \dot{\gamma } & =\frac{M_{z}}{I_{z}}, & \; \end{array}$$

where $I_{z}$ is the moment of inertia.

### 3.2. Load Transfer

A load transfer model is introduced to evaluate the tire forces when a vehicle undergoes significant changes in both longitudinal and lateral accelerations. Referring to [19], the equations for the static load transfer are

(15) $$\begin{array}{cc} F_{zfl} & =\frac{1}{2}m_{v}\left(\frac{l_{r}}{l}g-\frac{h_{c}}{l}{\bar{a}}_{x}\right)-m_{v}\left(\frac{l_{r}}{l}g-\frac{h_{c}}{l}{\bar{a}}_{x}\right)\frac{h_{c}}{w_{t}}\frac{{\bar{a}}_{y}}{g}-\frac{F_{\mathrm{lift}}}{4}, \end{array}$$

(16) $$\begin{array}{cc} F_{zfr} & =\frac{1}{2}m_{v}\left(\frac{l_{r}}{l}g-\frac{h_{c}}{l}{\bar{a}}_{x}\right)+m_{v}\left(\frac{l_{r}}{l}g-\frac{h_{c}}{l}{\bar{a}}_{x}\right)\frac{h_{c}}{w_{t}}\frac{{\bar{a}}_{y}}{g}-\frac{F_{\mathrm{lift}}}{4}, \end{array}$$

(17) $$\begin{array}{cc} F_{zrl} & =\frac{1}{2}m_{v}\left(\frac{l_{f}}{l}g+\frac{h_{c}}{l}{\bar{a}}_{x}\right)-m_{v}\left(\frac{l_{f}}{l}g+\frac{h_{c}}{l}{\bar{a}}_{x}\right)\frac{h_{c}}{w_{t}}\frac{{\bar{a}}_{y}}{g}-\frac{F_{\mathrm{lift}}}{4}, \end{array}$$

(18) $$\begin{array}{cc} F_{zrr} & =\frac{1}{2}m_{v}\left(\frac{l_{f}}{l}g+\frac{h_{c}}{l}{\bar{a}}_{x}\right)+m_{v}\left(\frac{l_{f}}{l}g+\frac{h_{c}}{l}{\bar{a}}_{x}\right)\frac{h_{c}}{w_{t}}\frac{{\bar{a}}_{y}}{g}-\frac{F_{\mathrm{lift}}}{4}, \end{array}$$

where *l* is the wheelbase, $h_{c}$ is the height of CG, *g* is the gravity constant, ${\bar{a}}_{x}$ and ${\bar{a}}_{y}$ are the decision variables provided by the NLP solver, while the aerodynamic lift force, characterized by the lift coefficient $C_{l}$, is given by $F_{\mathrm{lift}}=\frac{1}{2}\rho C_{l}AV_{x}^{2}$.

To prevent algebraic loop issues being encountered by the optimization solver during function evaluations, the algebraic variables ${\bar{a}}_{x}$ and ${\bar{a}}_{y}$ are introduced, and constraints are imposed to ensure their values are consistent with the vehicle accelerations (7) and (8),

(19) $$\begin{array}{cc} {\bar{a}}_{x}-a_{x} & =0, \end{array}$$

(20) $$\begin{array}{cc} {\bar{a}}_{y}-a_{y} & =0. \end{array}$$

To improve the convergence of online NMPC solvers, the equality constraints on accelerations are approximated using first-order dynamics as the following equations:

(21) $$\begin{array}{c} {\dot{\bar{a}}}_{x}=\frac{1}{\tau_{\mathrm{acc}}}\left(a_{x}-{\bar{a}}_{x}\right) \end{array}$$

(22) $$\begin{array}{c} {\dot{\bar{a}}}_{y}=\frac{1}{\tau_{\mathrm{acc}}}\left(a_{y}-{\bar{a}}_{y}\right) \end{array}$$

where the time constant $\tau_{\mathrm{acc}}$ is set to half the NMPC sampling time.

### 3.3. Wheel Torques

The torque distribution between the front and rear axles are determined by the following equations:

(23) $$\begin{array}{ccc} T_{f} & =k_{t}T_{t}+k_{b}T_{b}, & \; \end{array}$$

(24) $$\begin{array}{cc} T_{r} & =\left(1-k_{t}\right)T_{t}+\left(1-k_{b}\right)T_{b}, \end{array}$$

where $T_{t}$ and $T_{b}$ are the total traction torque and brake torque commands. $k_{b}$ denotes the distribution ratio of brake torque between the front and rear axles, while $k_{t}$ indicates the split coefficient for traction, which is determined by the drivetrain type of the vehicle. For the RWD vehicle considered in this study, $k_{t}=0$ and $k_{b}=0.6$.

The axle torques $T_{f}$ and $T_{r}$ are then redistributed to the left and right wheels in proportion to the relative vertical tire forces, similarly to the strategy in [20]. Then, the torque commands for the four wheel are determined by the following equations:

(25) $$\begin{array}{ccccc} T_{fl} & =\frac{T_{f}}{2}\left(1-\frac{\Delta F_{zf}}{F_{zf}}\right), & \; & T_{fr} & =\frac{T_{f}}{2}\left(1+\frac{\Delta F_{zf}}{F_{zf}}\right), \end{array}$$

(26) $$\begin{array}{ccccc} T_{rl} & =\frac{T_{r}}{2}\left(1-\frac{\Delta F_{zr}}{F_{zr}}\right), & \; & T_{rr} & =\frac{T_{r}}{2}\left(1+\frac{\Delta F_{zr}}{F_{zr}}\right), \end{array}$$

where the relative vertical loads are calculated as

(27) $$\begin{array}{cc} \Delta F_{zf} & =\frac{1}{2}\left(F_{zfr}-F_{zfl}\right)=m_{v}\left(\frac{l_{r}}{l}g-\frac{h_{c}}{l}{\bar{a}}_{x}\right)\frac{h_{c}}{w_{t}}\frac{{\bar{a}}_{y}}{g}, \end{array}$$

(28) $$\begin{array}{cc} \Delta F_{zr} & =\frac{1}{2}\left(F_{zrr}-F_{zrl}\right)=m_{v}\left(\frac{l_{f}}{l}g+\frac{h_{c}}{l}{\bar{a}}_{x}\right)\frac{h_{c}}{w_{t}}\frac{{\bar{a}}_{y}}{g}. \end{array}$$

### 3.4. Tire Modeling

According to the moment and force balance equations, the dynamics of wheel angular velocity are described by the following equation:

(29) $${\dot{\omega }}_{i}=\frac{1}{I_{w}}\left(T_{i}-r_{w}F_{xi}\right),\;i\in \left\{fl,fr,rl,rr\right\},$$

where $T_{i}$ is the torque applied on the wheel, $I_{w}$ is the spin moment of inertia of the wheel, and $r_{w}$ is the effective rolling radius.

The longitudinal velocities of the tire trajectories are given by

(30) $$\begin{array}{cc} V_{xfl} & =\left(V_{x}-\frac{w_{t}}{2}\gamma \right)\cos\left(\delta \right)+\left(V_{y}+l_{f}\gamma \right)\sin\left(\delta \right), \end{array}$$

(31) $$\begin{array}{cc} V_{xfr} & =\left(V_{x}+\frac{w_{t}}{2}\gamma \right)\cos\left(\delta \right)+\left(V_{y}+l_{f}\gamma \right)\sin\left(\delta \right), \end{array}$$

(32) $$\begin{array}{ccc} V_{xrl} & =V_{x}-\frac{w_{r}}{2}\gamma , & \; \end{array}$$

(33) $$\begin{array}{ccc} V_{xrr} & =V_{x}+\frac{w_{r}}{2}\gamma , & \; \end{array}$$

and the lateral velocities of the tire trajectories are given by

(34) $$\begin{array}{cc} V_{yfl} & =\left(V_{y}+l_{f}\gamma \right)\cos\left(\delta \right)-\left(V_{x}-\frac{w_{t}}{2}\gamma \right)\sin\left(\delta \right), \end{array}$$

(35) $$\begin{array}{cc} V_{yfr} & =\left(V_{y}+l_{f}\gamma \right)\cos\left(\delta \right)-\left(V_{x}+\frac{w_{t}}{2}\gamma \right)\sin\left(\delta \right), \end{array}$$

(36) $$\begin{array}{ccc} V_{yrl} & =V_{y}-l_{r}\gamma , & \; \end{array}$$

(37) $$\begin{array}{ccc} V_{yrr} & =V_{y}-l_{r}\gamma . & \; \end{array}$$

Then, the longitudinal slip λ and sideslip angle α of the tire at each corner $i\in \left\{fl,fr,rl,rr\right\}$ are defined by

(38) $$\begin{array}{cc} \lambda_{i} & =\frac{r_{w}\omega_{i}-V_{xi}}{V_{xi}}, \end{array}$$

(39) $$\begin{array}{cc} \alpha_{i} & =\tan^{-1}\left(\frac{V_{yi}}{V_{xi}}\right). \end{array}$$

#### 3.4.1. Simplified MF Tire Model

**Figure 3:**
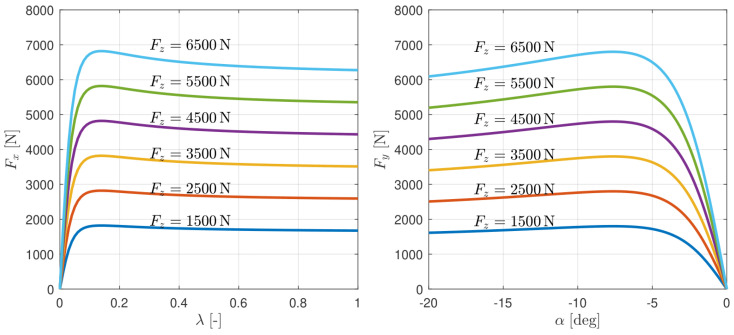
Simplified MF tire model.

The simplified magic formula (simplified MF) [21] (ch. 9.2.2, [22]) tire model is used to capture the nonlinear characteristics of tire forces. This model is easy to implement and has good continuity, providing advantages for optimization algorithms that require model gradients. The simplified MF for the longitudinal tire forces under pure longitudinal slip conditions is shown in the left plot of Figure 3. It is the function of the longitudinal tire slip λ, vertical tire force $F_{z}$, and road friction coefficient μ, as shown in the following equation:

(40) $$F_{x0}\left(\lambda ,F_{z},\mu \right)=\frac{\mu }{\mu_{0}}D_{x}\sin\left[C_{x}\tan^{-1}\left(B_{x}\lambda \right)\right],$$

where $\mu_{0}$ is the road friction coefficient during tire testing, and $B_{x}$, $C_{x}$, and $D_{x}$ are fitting coefficients. Since the peak factor $D_{x}$ varies linearly with the vertical tire force, it can be modeled as a first-order function of $F_{z}$ with a linear term $d_{1}$ and a constant term $d_{2}$,

(41) $$D_{x}=d_{1x}F_{z}+d_{2x}.$$

The simplified MF for the lateral tire forces under pure sideslip conditions is shown in the right plot of Figure 3, and it is expressed by the following equation:

(42) $$F_{y0}\left(\alpha ,F_{z},\mu \right)=-\frac{\mu }{\mu_{0}}D_{y}\sin\left[C_{y}\tan^{-1}\left(B_{y}\alpha \right)\right],$$

where

(43) $$D_{y}=d_{1y}F_{z}+d_{2y}.$$

#### 3.4.2. Combined Slip Tire Model for TRO

In highly dynamic scenarios, a combination of braking and cornering maneuvers is commonly encountered, leading to coupled lateral and longitudinal tire slips and forces. Consequently, the coupled slip effect is incorporated into the TRO, to provide an accurate reference trajectory. The combined slip model from (ch. 4.2.2, [22]) is employed to evaluate the tire forces under combined slip conditions, where the theoretical slip quantities at four corners are defined as

(44) $$\sigma_{i}=\sqrt{\lambda_{i}^{2}+\tan{\left(\alpha_{i}\right)}^{2}},\;i\in \left\{fl,fr,rl,rr\right\}.$$

And the combined slip longitudinal and lateral tire forces of are defined by

(45) $$\begin{array}{c} F_{xi}=\frac{\lambda_{i}}{\sigma_{i}}F_{x0}\left(\sigma_{i},F_{zi},\mu \right),\;F_{yi}=\frac{\tan(\alpha_{i})}{\sigma_{i}}F_{y0}\left(\sigma_{i},F_{zi},\mu \right),\;i\in \left\{fl,fr,rl,rr\right\}. \end{array}$$

#### 3.4.3. Pure Sideslip Tire Model for NMPC

Considering the convergence and real-time requirements of the online trajectory tracking task, wheel dynamics are not considered in the NMPC formulation. Instead, the combined tire force is characterized by tire ellipse constraints. Consequently, the longitudinal and lateral tire forces are described by the following equations:

(46) $$\begin{array}{c} F_{xi}=\frac{T_{i}}{r_{w}},\;F_{yi}=F_{y0}\left(\alpha_{i},F_{zi},\mu \right),\;i\in \left\{fl,fr,rl,rr\right\}. \end{array}$$

### 3.5. Vehicle Constraints

Quadratic nonlinear constraints are imposed to ensure that the combined tire forces remain within the tire friction ellipses, thereby preventing a loss of traction.

(47) $${\left(\frac{F_{xi}}{\mu_{x,\max}F_{zi}}\right)}^{2}+{\left(\frac{F_{yi}}{\mu_{y,\max}F_{zi}}\right)}^{2}\leq 1,\;i\in \left\{fl,fr,rl,rr\right\},$$

where $\mu_{x,\max}$ and $\mu_{y,\max}$ are the maximum longitudinal and lateral tire friction coefficients.

To achieve the optimal actuation efficiency, it is not desirable to apply both motor and brake torque to the wheel at the same time. Thus, an equality constraint is imposed to ensure that the total traction and braking commands are orthogonal:

(48) $$T_{t}T_{b}=0.$$

Considering rear dual electric motors, two equality equations are introduced to limit the motor powers separately,

(49) $$\begin{array}{cc} T_{rl}\omega_{rl} & \leq P_{rl,\max},\\ T_{rr}\omega_{rr} & \leq P_{rr,\max}, \end{array}$$

where $P_{\;\cdot \;,\max}$ is the maximum motor power for the respective motor, ω is the wheel angular velocity, and subscripts rl and rr denote the rear-left and rear-right wheels, respectively.

## 4. Control Architecture

**Figure 4:**
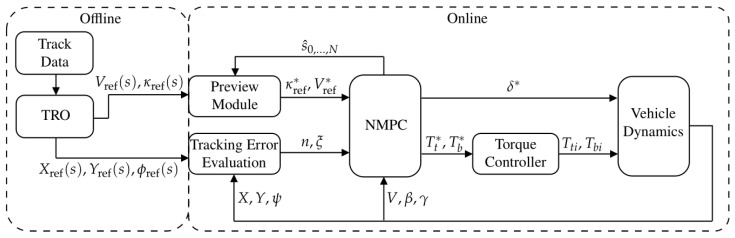
Control architecture for the path following problem.

The overall control architecture is shown in Figure 4, with modules for offline and online execution. The offline TRO module generates the time-optimal trajectory based on the racetrack data. The optimal curvature, velocity, and raceline trajectories are then provided to the online modules. During the online path tracking stage, the tracking error evaluation module calculates the lateral deviation and heading error using the algorithm proposed in [23]. The preview module queries the reference curvature and speed trajectories based on the preview path station. The NMPC module solves the optimal vehicle inputs for the online trajectory tracking. Subsequently, the low-level controller distributes the desired torque commands as rear driving torques and four-wheel braking torques.

## 5. Trajectory Optimization

Trajectory optimization aims to determine the reference path with the shortest travel time for a given racetrack, along with the corresponding vehicle states and input trajectories, while satisfying specified constraints. Prior to optimization, the centerline of the racetrack needs to be smoothed and discretized into uniform grid distances. Subsequently, the direct orthogonal collocation method is employed to discretize the system dynamics and cost functions in the spatial domain, thereby transforming the optimal control problem (OCP) into a standard NLP formulation.

### 5.1. System Dynamics in the Spatial Domain

The TRO problem is formulated in the spatial domain, using the distance along the track as the independent variable instead of time. This allows the dynamics to be expressed as a function of the path position, rather than time. The state variables of the TRO are concatenated from the vehicle states, wheel velocities, and path tracking states,

(50) $$x={\left[\begin{array}{ccccccccc} V & \beta & \gamma & \omega_{fl} & \omega_{fr} & \omega_{rl} & \omega_{rr} & n & \xi \end{array}\right]}^{⊤}.$$

The control variables are the total traction/braking torque commands and front wheel steering angle,

(51) $$u={\left[\begin{array}{ccc} T_{t} & T_{b} & \delta \end{array}\right]}^{⊤}.$$

The auxiliary variables are the algebraic longitudinal and lateral accelerations,

(52) $$z={\left[\begin{array}{cc} {\bar{a}}_{x} & {\bar{a}}_{y} \end{array}\right]}^{⊤}.$$

Then, the system dynamics are the function of state, control, auxiliary and parameter variables, as shown in the following equations:

(53) $$\begin{array}{cc} \dot{x} & ={\left[\begin{array}{cccccccccccccc} \dot{V} & \; & \dot{\beta } & \; & \dot{\gamma } & \; & {\dot{\omega }}_{fl} & {\dot{\omega }}_{fr} & \; & {\dot{\omega }}_{rl} & {\dot{\omega }}_{rr} & \; & \dot{n} & \;\dot{\xi } \end{array}\right]}^{⊤}\\ & ={\left[\begin{array}{ccccccccc} \left(12\right) & \left(13\right) & \left(14\right) & \left(29\right) & \left(29\right) & \left(29\right) & \left(29\right) & \left(1\right) & \left(3\right) \end{array}\right]}^{⊤}. \end{array}$$

The system dynamics in the spatial domain $x^{\prime }$ are transformed from the time domain using the variable $S_{f}$,

(54) $$\begin{array}{c} x^{\prime }=\frac{dx}{ds}=\frac{dt}{ds}\frac{dx}{dt}≕S_{f}\dot{x}f_{s}\left(x,u,z,\kappa \right). \end{array}$$

The magnitudes of states, controls, and auxiliary variables are bounded due to the physical limits of the vehicle and actuators,

(55) $$\begin{array}{cc} x_{\min} & \leq x\leq x_{\max}, \end{array}$$

(56) $$\begin{array}{cc} u_{\min} & \leq u\leq u_{\max}, \end{array}$$

(57) $$\begin{array}{cc} z_{\min} & \leq z\leq z_{\max}, \end{array}$$

where the subscripts min and max denote the lower and upper bounds of the variables.

The rates of the control variables are limited according to the dynamic characteristics of the actuators,

(58) $$\begin{array}{cc} \; & {\dot{u}}_{\min}\leq \dot{u}\leq {\dot{u}}_{\max}. \end{array}$$

### 5.2. Direct Collocation

**Figure 5:**
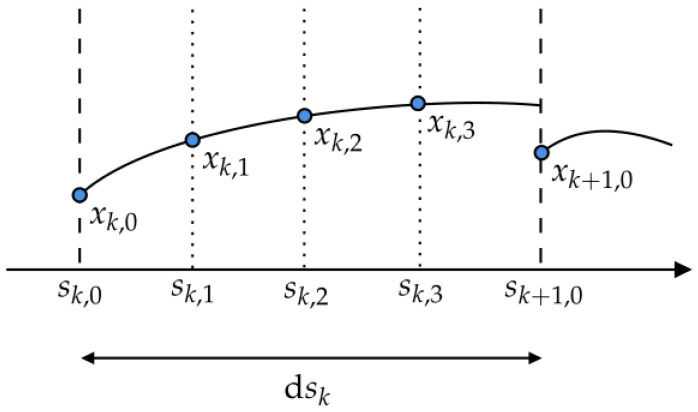
Illustration of the decision variables for the direct collocation with q=3 in a path interval.

As Figure 5 shows, the distance along the track centerline $\left[s_{0},s_{f}\right]$ is divided into *N* intervals by the step length $ds_{k}$, with each interval spanning $\left[s_{k},s_{k+1}\right]$, where *k* ranges from 0 to N−1.

The state trajectory of the interval is approximated using the Legendre polynomial as the following equation:

(59) $$x_{k}\left(\tau \right)=\sum_{i=0}^{q}P_{i}\left(\tau \right)x_{k,i},\;i=0,\ldots ,q.$$

where $P_{i}\left(\tau \right)$ is the basis of the Legendre polynomial over the unit interval τ=[1,0], computed using Gauss–Legendre collocation points $\left\{\tau_{0},\tau_{1},\ldots ,\tau_{q}\right\}$ (ch. 10.3, [24]) with order *q*, as defined by

(60) $$P_{i}\left(\tau \right)=\prod_{\begin{array}{c} \;\;j=0,\\ j\neq i \end{array}}^{q}\frac{\tau -\tau_{j}}{\tau_{i}-\tau_{j}},\;i=0,\ldots ,q.$$

To facilitate the representation of state trajectories within each interval for subsequent formulations, the state variables at the knot and collocation points are aggregated into a matrix denoted as $X_{k}$,

(61) $$X_{k}=\left[\begin{array}{cccc} x_{k,0} & x_{k,1} & \cdots & x_{k,q} \end{array}\right]\in R^{n_{x}\times \left(q+1\right)},\;k=0,\ldots ,N-1.$$

The direct collocation method is utilized to discretize the TRO problem, following the approaches outlined in [11,25,26]. This transcription method involves imposing additional constraints to enforce the polynomials approximate to the system dynamics.

For each interval, the derivatives of Legendre polynomials at the collocation points must match the system dynamics, as in the following collocation equation:

(62) $$X_{k}C-ds_{k}\left[\begin{array}{ccc} x_{k,1}^{\prime }\; & \cdots \; & x_{k,q}^{\prime } \end{array}\right]=0,\;k=0,\ldots ,N-1,$$

where $x_{k,j}^{\prime }=f_{s}\left(x_{k,j},u_{k},z_{k},\kappa_{k}\right)$ are the system dynamics in the spatial domain at the collocation points, and the control $u_{k}$ is piece-wise constant for $\left[s_{k},s_{k+1}\right)$.

The state trajectory at the end of the interval must be consistent with the beginning of the next interval, as seen in the following continuity equation:

(63) $$X_{k}D-x_{k+1}=0,\;k=0,\ldots ,N-1.$$

The matrix C is defined using the derivatives of the Legendre polynomials evaluated at collocation points, and the matrix D is defined using the Legendre polynomials evaluated at the end points of the interval,

(64) $$C=\left[\begin{array}{ccc} {\dot{P}}_{0}\left(\tau_{1}\right) & \cdots & {\dot{P}}_{0}\left(\tau_{q}\right)\\ \vdots & \ddots & \vdots \\ {\dot{P}}_{q}\left(\tau_{1}\right) & \cdots & {\dot{P}}_{q}\left(\tau_{q}\right) \end{array}\right]\in R^{\left(q+1\right)\times q},\;D=\left[\begin{array}{c} P_{0}\left(1\right)\\ \vdots \\ P_{q}\left(1\right) \end{array}\right]\in R^{q+1}.$$

Additional constraints set h include the tire ellipse, actuator orthogonality, motor power, and state/control limits, which are imposed at the knot points only,

(65) $$\begin{array}{c} h_{\min}\leq h\left(x_{k},u_{k},z_{k},\kappa_{k}\right)\leq h_{\max},\;k=0,\ldots ,N, \end{array}$$

where $h_{\min}$ and $h_{\max}$ are the lower and upper bounds of constraint.

The variation in control is mapped onto the time domain in a manner that aligns with the specified bounds within the time domain.

(66) $$\begin{array}{c} \dot{u}\approx \frac{du}{ds}\frac{ds}{dt}=u^{\prime }\dot{s}\;\to \;{\dot{u}}_{\min}\leq u^{\prime }\dot{s}\leq {\dot{u}}_{\max}. \end{array}$$

### 5.3. Cost Function

The cost function is an integral part of trajectory optimization, representing the objective to be minimized. To minimize the lap time, the time cost within each discretized path interval must be incorporated, as in the following equation:

(67) $$dt_{k}=ds_{k}\left[\begin{array}{ccc} S_{f}\left(x_{k,1},\kappa_{k}\right)\; & \cdots \; & S_{f}\left(x_{k,q},\kappa_{k}\right) \end{array}\right]B,\;k=0,\ldots ,N-1,$$

where the matrix B aggregates the contributions of the time cost at the collocation points of intervals,

(68) $$B={\left[\begin{array}{ccc} \int_{0}^{1}P_{1}\left(\tau \right)d\tau & \cdots & \int_{0}^{1}P_{q}\left(\tau \right)d\tau \end{array}\right]}^{⊤}\in R^{q}.$$

To obtain the shortest laptime with smooth vehicle inputs, the cost function sums the time cost and the quadratic cost of variations in control and auxiliary variables over all intervals,

(69) $$J_{\mathrm{TRO}}=\sum_{k=0}^{N-1}\left(dt_{k}+{u_{k}^{\prime }}^{⊤}Ru_{k}^{\prime }+{z_{k}^{\prime }}^{⊤}Wz_{k}^{\prime }\right),$$

where R and W are the weighting matrices, and if it is observed that the resulting optimal input commands exhibit oscillatory behavior, then the corresponding weights in the matrices need to be incremented. The variations in variables $u_{k}^{\prime }$ and $z_{k}^{\prime }$ are calculated using the following difference equations:

(70) $$\begin{array}{c} u_{k}^{\prime }=\frac{u_{k+1}-u_{k}}{ds_{k}},\;z_{k}^{\prime }=\frac{z_{k+1}-z_{k}}{ds_{k}},\;k=0,\ldots ,N-1. \end{array}$$

### 5.4. NLP Solver

To improve the convergence of the NLP, it is necessary to properly scale the decision variables of the solver [27]. The state and control variables are scaled by the scaling factors, which are determined by their maximum expected values. This normalization process ensures that all the variables lie within a common range, typically between −1 and 1. The detailed workflow and implementation of the scaling procedure can be found in [28].

The overall TRO problem is summarized in Table 1 and includes decision variables, constraints, lower and upper bounds, and scaling factors. The symbolic framework CasADi [29] is used to formulate the TRO problem and transform it into a NLP. The large-scale nonlinear optimization solver Ipopt [30] is used to solve the NLP.

## 6. Nonlinear Model Predictive Control

The NMPC serves as the online trajectory tracking controller of the hierarchical control architecture. Its primary objective is to solve the optimal driving inputs, to control the vehicle in following the time-optimal reference trajectories generated by the TRO layer in real time.

### 6.1. Prediction Model

The NMPC needs to predict the future vehicle states, inputs, path station, and tracking errors; thus, the state variables of prediction model are selected as

(71) $$x={\left[\begin{array}{ccccccccccc} V & \beta & \gamma & {\bar{a}}_{x} & {\bar{a}}_{y} & s & n & \xi & T_{t} & T_{b} & \delta \end{array}\right]}^{⊤}.$$

The control variables are the rates of driving inputs:

(72) $$u=\left[\begin{array}{ccc} {\dot{T}}_{t,\mathrm{cmd}} & {\dot{T}}_{b,\mathrm{cmd}} & {\dot{\delta }}_{\mathrm{cmd}} \end{array}\right].$$

The system dynamics of the prediction model are formulated as:

(73) $$\begin{array}{cc} \dot{x} & ={\left[\begin{array}{ccccccccccccccccccccccc} \; & \dot{V} & \; & \dot{\beta } & \; & \dot{\gamma } & \; & {\dot{\bar{a}}}_{x} & \; & {\dot{\bar{a}}}_{y} & \; & \dot{s} & \; & \dot{n} & \; & \dot{\xi } & \; & {\dot{T}}_{t} & \; & {\dot{T}}_{b} & \; & \; & \dot{\delta } \end{array}\right]}^{⊤}\\ & ={\left[\begin{array}{ccccccccccc} \left(12\right) & \left(13\right) & \left(14\right) & \left(21\right) & \left(22\right) & \left(4\right) & \left(1\right) & \left(3\right) & {\dot{T}}_{t,\mathrm{cmd}} & {\dot{T}}_{t,\mathrm{cmd}} & {\dot{T}}_{t,\mathrm{cmd}} \end{array}\right]}^{⊤}\\ & ≕f_{p}\left(x,u,\kappa \right). \end{array}$$

The first to fifth equations in (73) predict the vehicle states and tire vertical loads, the sixth to eighth equations predict the path tracking errors and driving distance. In the last three equations, we assign the rate of system inputs as the extended states, similarly to [5,15,31]. Using the variation in driving inputs as the control variable allows us to conveniently penalize it when formulating optimization problems at a higher interface level, thereby aligning with the cost function of TRO (69).

The magnitudes of states, controls, and rates of the control variables are bounded, due to the physical limits of the vehicle and actuators,

(74) $$\begin{array}{cc} x_{\min} & \leq x\leq x_{\max}, \end{array}$$

(75) $$\begin{array}{cc} u_{\min} & \leq u\leq u_{\max}, \end{array}$$

(76) $$\begin{array}{cc} {\dot{u}}_{\min} & \leq \dot{u}\leq {\dot{u}}_{\max}. \end{array}$$

### 6.2. Discretization

The implicit Runge–Kutta method (IRK) of second order is used to discretize the prediction model over the horizon $k=0,\ldots ,N_{p}-1$,

(77) $$\begin{array}{cc} x_{0} & =\hat{x}\left(t\right), \end{array}$$

(78) $$\begin{array}{cc} x_{k+1} & =x_{k}+\frac{t_{s}}{2}\left[f_{p}\left(x_{k},u_{k},\kappa_{k}\right)+f_{p}\left(x_{k+1},u_{k+1},\kappa_{k+1}\right)\right], \end{array}$$

where $\hat{x}\left(t\right)$ is the measured or estimated stated at the current time, and $t_{s}=0.05\;s$ is the sample time of NMPC or the time interval for solving each optimization problem. The prediction horizon is set as $N_{p}=30$, resulting in the prediction time $t_{p}=N_{p}t_{s}=1.5\;s$.

### 6.3. Preview Path Station

In this section, an algorithm is proposed for generating the optimal reference trajectory for NMPC based on the preview path station. The preview path stations are obtained by moving the previous optimal path station by one step and adding a correction term to predict the distance to move for an additional step. Specifically, the preview path station ${\hat{s}}_{k}$ at each stage *k* is set equal to the previous optimal path station:

(79) $$\begin{array}{c} {\hat{s}}_{k}=s_{k+1}^{*},\;k=0,\ldots ,N_{p}-1, \end{array}$$

This ensures that the NMPC tracks the most recent optimal path station.

For the final preview path station ${\hat{s}}_{N_{p}}$, a correction term is added to extrapolate the distance the vehicle is expected to travel in the final prediction step:

(80) $$\begin{array}{c} {\hat{s}}_{N_{p}}=s_{k+1}^{*}+t_{s}V_{N_{p}}^{*}, \end{array}$$

where $V_{N_{p}}^{*}$ is the optimal velocity at the end of the prediction horizon, obtained from the previous NMPC solution. By interpolating the reference trajectory planned by TRO using the preview path stations that adapted to the vehicle’s current velocity, accurate preview references can be obtained for the NMPC to track.

### 6.4. Reference Output

The output of NMPC is $y={\left[V,\;\beta ,\;n,\;\chi \right]}^{⊤}$, and the reference output at each stage is given by

(81) $$\begin{array}{c} y_{k,\mathrm{ref}}={\left[\begin{array}{cccc} V_{\mathrm{ref}}\left({\hat{s}}_{k}\right) & \tan^{-1}\left(\delta \frac{l_{r}}{l}\right) & 0 & 0 \end{array}\right]}^{⊤}, \end{array}$$

meaning that the vehicle is expected to follow the reference velocity with zero path tracking errors, and the vehicle slip angle follows the kinematic reference value to maintain lateral stability.

### 6.5. Cost Function

Model–plant mismatches or external disturbances can lead to constraint violations that render the original optimization problem infeasible. To address this issue, a slack variable $s_{h}$ is introduced to handle constraint violations, while still maintaining a feasible solution,

(82) $$\begin{array}{c} h_{\min}-s_{h}\leq h\leq h_{\max}+s_{h}. \end{array}$$

where h includes the actuator orthogonality, four tire ellipse constraints, and power limits.

To balance the objectives of accurately tracking the desired trajectory, minimizing control efforts, and permitting constraint relaxation, the cost function to be minimized over the prediction horizon $N_{p}$ is formulated by combining tracking errors, control efforts, and penalties on the slack variables:

(83) $$J_{\mathrm{NMPC}}=\sum_{k=1}^{N_{p}}\frac{1}{2}{S_{y}^{-1}\left(y_{k}-y_{k,\mathrm{ref}}\right)}_{Q}^{2}+\sum_{k=0}^{N_{p}-1}\frac{1}{2}{S_{u}^{-1}u_{k}}_{R}^{2}+\frac{1}{2}s_{h}^{⊤}Zs_{h}\;,$$

where $S_{y}$ and $S_{u}$ are the scaling matrices, utilizing the maximum allowable deviation from the nominal value as the scaling factors. The weighting matrices Q, R, and Z penalize tracking errors, control efforts, and the slack variable, respectively. The elements within the Q matrix are adjusted to prioritize varying objectives, such as improving velocity tracking or decreasing lateral deviation. Similarly, the R matrix must be fine-tuned to prevent oscillatory behavior in the driving inputs.

The overall NMPC path tracking problem is formulated in Table 2. The fast and embedded solvers acados [32] was utilized as the NMPC solver, which features the implicit Runge–Kutta (IRK) for numerical simulation and the multiple shooting approach to discretize the OCP. Real-time iteration was also implemented, to perform one sequential quadratic programming (SQP) iteration at each sampling step, to further reduce the NMPC computation time. The OCP formulation, integrators, transcription methods, weights, parameters, and QP solvers were stated at the high-level interface of Matlab. In addition, code generation was used to automatically generate a fast NMPC C code that was deployed in Simulink via the S-function. The solver parameters are specified in Table A2.

## 7. Numerical Results

### 7.1. Simulation Setup

The simulations were performed on a desktop computer with an Intel^®^ i5-12500 processor. The online modules of Figure 4 were implemented and run in Simulink. The simulation was demonstrated on a lap of the Catalunya Circuit, with the raw data of track being extracted from the racetrack-database (https://github.com/TUMFTM/racetrack-database, accessed on 5 October 2024) and the time-optimal trajectories generated by the proposed TRO algorithm. The total length of the raceline $s_{f}=4584\;m$ was divided into uniform discrete intervals of ds=3m for TRO planning and ds=1m for NMPC tracking. To examine the convergence of NMPC at low speeds, where the numerical sensitivity to small longitudinal velocity and its associated gradients can be issues, the initial speed of the vehicle model in TRO, NMPC, and Simulink were set to $1\;{m\;s}^{-1}$.

### 7.2. Path Tracking Results

**Figure 6:**
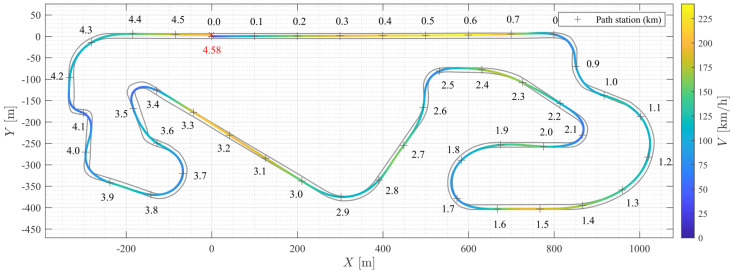
Path tracking response in global coordinates.

Figure 6 presents the path tracking response in global coordinates. The raceline displays the position of the vehicle’s CG with the velocity contour. It can be observed that the vehicle decelerated appropriately before reaching the apex, accelerated during the exit phase, and maintained high speeds on the straight sections of the track. The average velocity of the vehicle was $116.5\;km\;h^{-1}$. The lap time achieved by the NMPC was 141.45s, which was 1.37s more than the optimal lap time solved by the TRO, representing a relative error of 0.98%.

**Figure 7:**
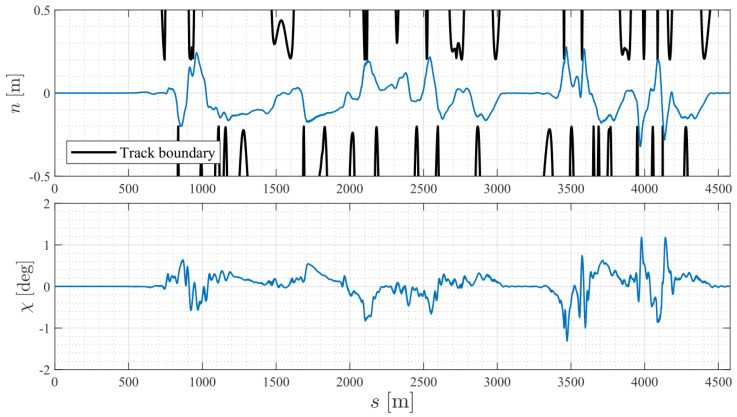
Path tracking errors in Cartesian coordinates.

Figure 7 presents the path tracking errors in Cartesian coordinates. The NMPC considered the future vehicle states, path tracking states, and preview references to coordinate the vehicle lateral and longitudinal motions, allowing the vehicle to follow the reference path with minimal tracking errors. Under the imposed racetrack constraints, the vehicle remained within the track boundaries during the race, as is evident from the blue line representing the lateral deviation not reaching the black line boundaries. The RMS and maximum values of the absolute lateral deviation were 0.11m and 0.32m, respectively. The RMS and maximum values of the absolute course error were 0.33deg and 1.28deg, respectively.

### 7.3. Vehicle States

**Figure 8:**
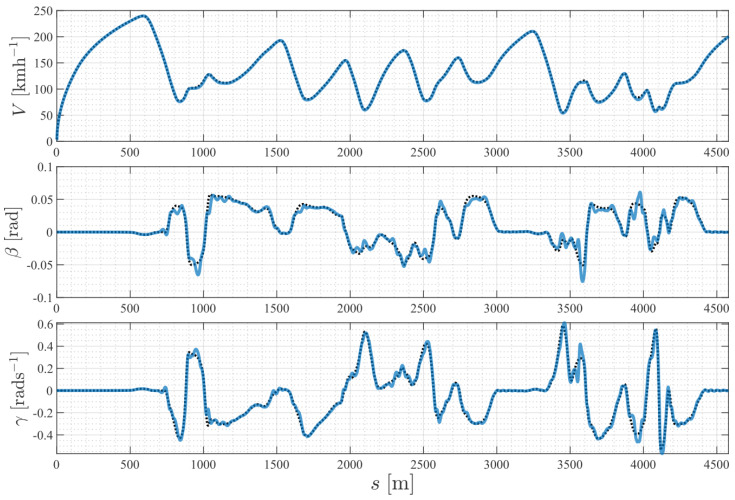
Trajectory tracking results with vehicle states. The black dotted lines are the optimal trajectories computed by the TRO. The blue lines are the tracking results achieved by the NMPC.

Figure 8 demonstrates the performance of the NMPC controller in accurately tracking the highly dynamic trajectories planned by the TRO layer. The NMPC controlled the vehicle to approach the at-limit speed profile, while utilizing the yaw rate to follow an aggressive path curvature, and keeping the sideslip angle within a stable range.

### 7.4. Driving Inputs

**Figure 9:**
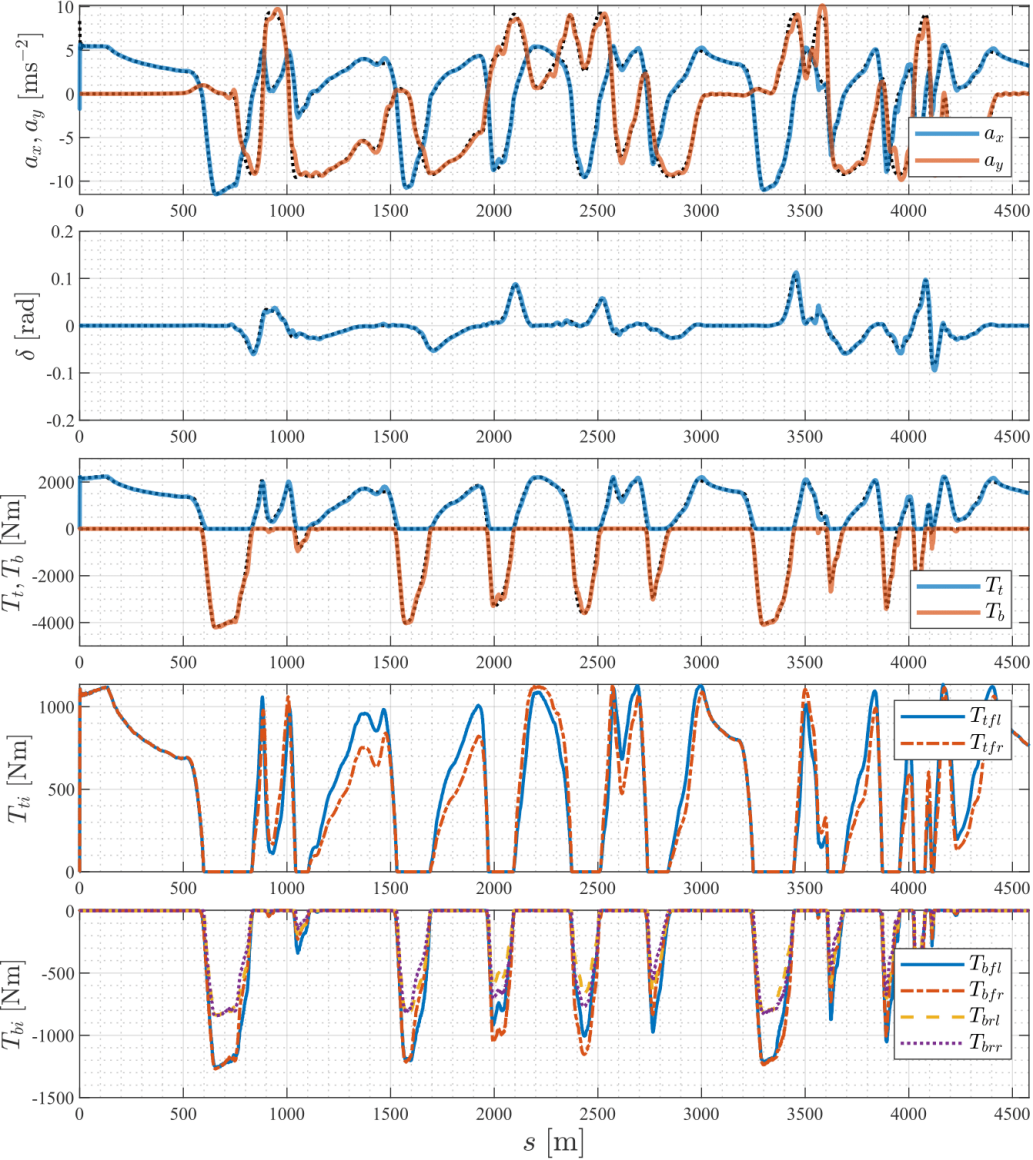
Optimal driving inputs and wheel torque commands. The black dotted lines are the optimal trajectories computed by the TRO.

Figure 9 shows the optimal vehicle accelerations, driving inputs, and wheel torque commands. The first plot shows that the vehicle acceleration solved by the NMPC controller was consistent with the planned acceleration from the TRO, exhibiting a similar trend in the torque commands. The steering and total torque commands were smooth, without any oscillation, which made them easier for the actuators to implement, and the total traction and braking commands satisfied the orthogonal constraint, enabling the actuators to operate with optimal efficiency. The traction and braking torques were appropriately distributed to four wheels based on the vertical load of each wheel.

### 7.5. Tire Workloads

**Figure 10:**
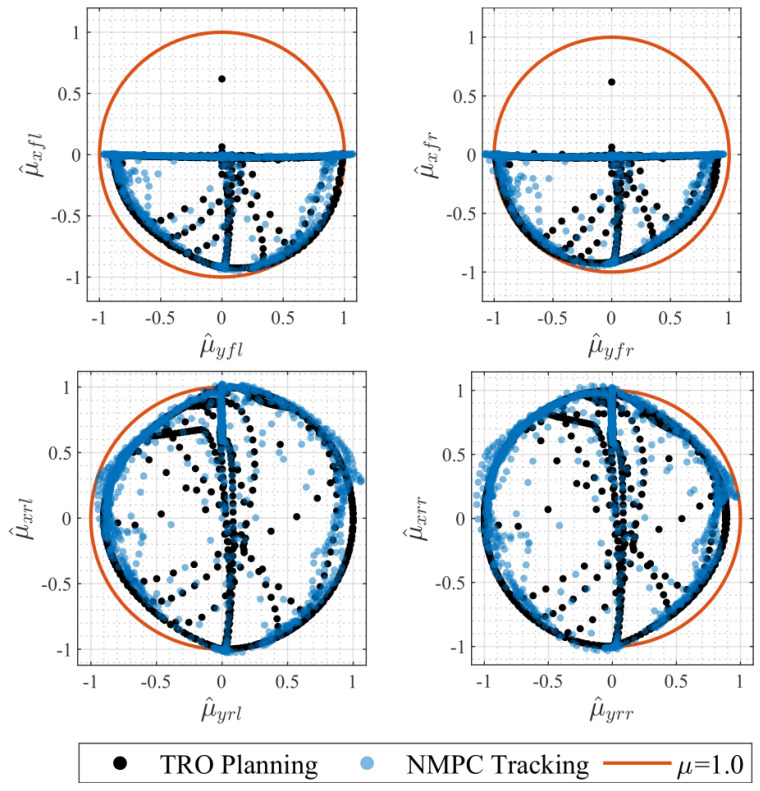
Tire workloads.

Figure 10 shows the tire grip margin using the tire workload plots, where the tire workloads in longitudinal and lateral directions are defined by

(84) $${\hat{\mu }}_{xi}=\frac{F_{xi}}{F_{zi}},\;{\hat{\mu }}_{yi}=\frac{F_{yi}}{F_{zi}},\;i\in \left\{fl,fr,rl,rr\right\}.$$

Under the tire ellipse constraint, the majority of the tire friction data points for all four wheels lay within the friction circle. The data points exceeding the boundary constituted 16% and 13% of the total data points for the left-rear wheel and right-rear wheel, respectively. The maximum values of the tire workloads did not exceed μ=1.1, which falls within an acceptable tolerance range.

## 8. Execution Performance

**Figure 11:**
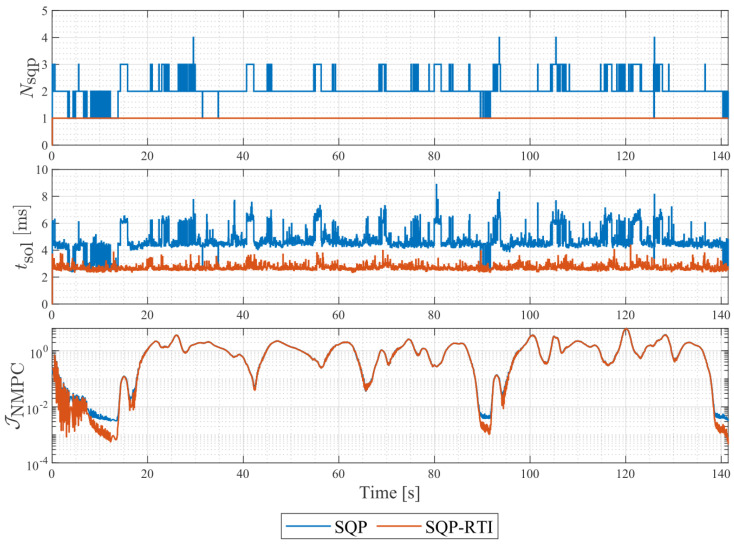
SQP iteration, execution time, and residual of cost function of NMPC solver.

Figure 11 records information about the acados solver. We configured the solver with both the SQP and SQP-RTI schemes and performed racing simulations, while keeping the other parameters unchanged. Both schemes achieved the same lap time and consistent cost function residuals. However, there was a significant difference in the computation time: the average computation time per sampling instance was 0.01 s for SQP and 0.005 s for RTI. It is noteworthy that the number of iterations using the SQP scheme alone did not exceed five, indicating that the problem exhibited good convergence and was well-defined. This provided a suitable environment for the RTI scheme, which performed only one iteration per sampling time. Furthermore, the cost function residuals being close to unity suggests that all the decision variables of the solver were appropriately scaled.

## 9. Conclusions

The proposed TOTPT framework utilizes a two-layered approach, with an offline TRO module and an online NMPC module. The TRO layer generates minimum-lap-time trajectories using a direct collocation method, which optimizes the vehicle’s path, velocity, and control inputs to achieve the fastest possible lap time, while respecting vehicle dynamics and track constraints. This computationally expensive offline optimization leverages a consistent double-track vehicle model to ensure coherence between the planning and tracking stages. The NMPC layer, operating in real time, is responsible for precisely tracking the reference trajectories generated by the TRO. By incorporating predicted path stations and adapting the reference trajectory based on the vehicle’s current speed and projected travel distance, the NMPC controller is able to maintain accurate path following, even under highly dynamic conditions. Notably, the NMPC formulation achieved millisecond-level execution times through the use of the RTI scheme. The authors validated the TOTPT framework through simulation on the Catalunya circuit, where the vehicle was able to track the optimal racing line with high speed and low tracking errors. The results highlight the framework’s ability to push the vehicle to its limits, which could translate to enhanced maneuverability and safety for autonomous vehicles in emergency scenarios on public roads.

### Future Work

Although the proposed control scheme demonstrated precise tracking results in the simulation, certain aspects require further improvement for practical application in future work. Firstly, while the simulation results demonstrated the framework’s performance in controlling a planar vehicle model on a two-dimensional track, it would be valuable to evaluate its robustness and adaptability on tracks with different features (e.g., track curvature, elevation changes, surface conditions). Secondly, considering real-time path planning, we could either look for solvers capable of solving NLP in real-time or simplify the TRO problem by employing a point-mass vehicle model with g-g-v diagram constraints. Finally, conducting experiments in real environments is crucial. A viable approach would be to implement the algorithm in the low-cost autonomous vehicle system of the F1TENTH platform [33], conducting path-following experiments to evaluate the real-time performance and robustness of the control algorithms under practical conditions.

## Figures and Tables

**Table 1:** TRO formulation.

	Description	Symbol/ Equation	Scale	Lower	Upper	Units
**Minimize**	**Cost**	$J_{\mathrm{TRO}}$ (69)				
**By varying**						
States	Velocity	*V*	100	0	250/3.6	m s^−1^
	Sideslip angle	β	1	−π/4	π/4	rad
	Yaw rate	γ	1	−π/2	π/2	rad s^−1^
	Wheel velocities	$\omega_{i}$	$100/r_{w}$	0	$250/3.6/r_{w}$	rad s^−1^
	Lateral displacement	*n*	5	$-N_{r}+\left(w_{t}/2+w_{s}\right)$	$N_{l}-\left(w_{t}/2+w_{s}\right)$	m
	Relative heading	ξ	1	−π/4	π/4	rad
Controls	Traction torque	$T_{t}$	$2\;\times \;10^{3}$	0	$4\;\times \;10^{3}$	N m
	Braking torque	$T_{b}$	$4\;\times \;10^{3}$	$-8\;\times \;10^{3}$	0	N m
	Wheel steering angle	δ	π/8	−π/8	π/8	rad
Aux. variables	Longitudinal acceleration	${\bar{a}}_{x}$	*g*	−3g	3g	m s^−2^
	Lateral acceleration	${\bar{a}}_{y}$	*g*	−3g	3g	m s^−2^
Parameter	Path curvature	κ	–	–	–	m^−1^
**Subject to**						
Constraints	Dynamics as collocation	(62), (63)	–	–	–	–
	Equality of accelerations	(19),(20)	*g*	$-10^{-3}$	$10^{-3}$	–
	Tire ellipse	(47)	1	0	1	–
	Actuator orthogonality	(48)	1	$-10^{-3}$	$10^{-3}$	–
	Motor power limitation	(49)	$2\times 10^{5}/r_{w}$	0	150	kW
Bounds on	Rate of traction torque	${\dot{T}}_{t}$	$2\;\times \;10^{3}$	0	$3\;\times \;10^{3}$	N m s^−1^
	Rate of braking torque	${\dot{T}}_{b}$	$4\;\times \;10^{3}$	$-6\;\times \;10^{3}$	0	N m s^−1^
	Rate of wheel steering angle	$\dot{\delta }$	π/8	−π/8	π/8	rad s^−1^

**Table 2:** NMPC formulation.

	Description	Symbol/ Equation	Scale	Lower	Upper	Units
**Minimize**	**Cost**	$J_{\mathrm{NMPC}}$ (83)				
**By varing**						
States	Velocity	*V*	100	0	250/3.6	m s^−1^
	Sideslip angle	β	1	−π/4	π/4	rad
	Yaw rate	γ	1	−π/2	π/2	rad s^−1^
	Longitudinal acceleration	${\bar{a}}_{x}$	*g*	−3g	3g	m s^−2^
	Lateral acceleration	${\bar{a}}_{y}$	*g*	−3g	3g	m s^−2^
	Path station	*s*	1	0	Inf	m
	Lateral displacement	*n*	5	$-N_{r}+w_{t}/2$	$N_{l}-w_{t}/2$	m
	Relative heading	ξ	1	−π/4	π/4	rad
	Traction torque	$T_{t}$	$2\;\times \;10^{3}$	0	$4\;\times \;10^{3}$	N m
	Braking torque	$T_{b}$	$4\;\times \;10^{3}$	$-8\;\times \;10^{3}$	0	N m
	Wheel steering angle	δ	π/8	−π/8	π/8	rad
Controls	Rate of traction torque	${\dot{T}}_{t,\mathrm{cmd}}$	$2\;\times \;10^{3}$	0	$3\;\times \;10^{3}$	N m s^−1^
	Rate of braking torque	${\dot{T}}_{b,\mathrm{cmd}}$	$4\;\times \;10^{3}$	$-6\;\times \;10^{3}$	0	N m s^−1^
	Rate of wheel steering angle	${\dot{\delta }}_{\mathrm{cmd}}$	π/8	−π/8	π/8	rad s^−1^
Parameter	Path curvature	κ	–	–	–	m^−1^
Subjectto						
Constraints	Dynamics as IRK	(78)	–	–	–	–
	Initial condition	(77)	–	–	–	–
	Tire ellipse	(47)	1	0	1	–
	Actuator orthogonality	(48)	0.02	$-10^{-3}$	$10^{-3}$	–

## Data Availability

Data are contained within the article.

## Appendix A

**Table A1:** Parameters of the vehicle model used in the study.

	Description	Symbol	Value	Unit
**Env.**	gravity constant	*g*	9.81	$\;[m\;s^{-2}]$
	coefficient of road friction	$\mu_{0}$	1.0	–
	air density	ρ	1.2	$\;[kg\;m^{-3}]$
	coefficient of drag	$C_{d}$	0.3	–
	coefficient of lift	$C_{l}$	−0.6	–
**Vehicle**	front cross sectional area	*A*	1.5	$\;[m^{2}]$
	vehicle mass	$m_{v}$	1250	[kg]
	vehicle moment of inertia	$I_{z}$	1050	$\;[\mathrm{kg}\;m^{2}]$
	distance from CG to front axle	$l_{f}$	1.4	[m]
	distance from CG to rear axle	$l_{r}$	1.4	[m]
	height of CG	$h_{c}$	0.35	[m]
	track width	$w_{t}$	1.5	[m]
	wheel spin moment of inertia	$I_{w}$	1.2	$\;[\mathrm{kg}\;m^{2}]$
	wheel effective rolling radius	$r_{w}$	0.3	[m]
**Tire**	long. stiffness factor	$B_{x}$	18	–
	long. shape factor	$C_{x}$	1.3	–
	long. peak factor linear term	$d_{1x}$	0.95	–
	long. peak factor constant term	$d_{2x}$	320	–
	lateral stiffness factor	$B_{y}$	13	–
	lateral shape factor	$C_{y}$	1.4	–
	lateral peak factor linear term	$d_{1y}$	0.95	–
	lateral peak factor constant term	$d_{2y}$	320	–
	maximum longitudinal friction coeff.	$\mu_{x,\max}$	1.0	–
	maximum lateral friction coeff.	$\mu_{y,\max}$	1.0	–

**Table A2:** Parameters of the acados solver used in the NMPC implementation.

Option	Value
sim_method	irk
sim_method_num_stages	2
sim_method_num_steps	1
nlp_solver_max_iter	30
nlp_solver_tol_stat	$10^{-4}$
nlp_solver_tol_eq	$10^{-4}$
nlp_solver_tol_ineq	$10^{-4}$
nlp_solver_tol_comp	$10^{-4}$
qp_solver	full_condensing_hpipm
nlp_solver_exact_hessian	true
**Parameter**	
$S_{y}$	$\mathrm{diag}\left(1,\;0.05,\;0.1,\;0.05\right)$
$S_{u}$	$\mathrm{diag}\left(2\;\times \;10^{3},\;4\;\times \;10^{3},\;\frac{\pi }{8}\right)$
Q	$\mathrm{diag}\left(1,\;1,\;1,\;1\right)$
R	$\mathrm{diag}\left(1,\;1,\;10\right)$
Z	$\mathrm{diag}\left(10,\;10,\;10,\;10,\;10\right)$
